# Plaie isolée du cholédoque post traumatique, lésion inhabituelle: à propos d'un cas

**DOI:** 10.11604/pamj.2020.35.77.16703

**Published:** 2020-03-17

**Authors:** Yacoub Mohamed Sghair, Mohamedou Elhoucein, Ahmed Kane, Elhaj Adda, Mohamed Sidahmed, Mohamed Yeslem Mohamed Abdi, Sidi Med Naji

**Affiliations:** 1 Service de Chirurgie Pédiatrique, Centre Hospitalier Mère et Enfant, Faculté de Médecine de Nouakchott, Nouakchott, Mauritanie; 2 Service de Chirurgie Pédiatrique, Centre Hospitalier Mère et Enfant, Nouakchott, Mauritanie; 3 Service de Réanimation Anesthésie, Centre Hospitalier Mère et Enfant, Faculté de Médecine de Nouakchott, Nouakchott, Mauritanie; 4 Service de Radiologie, Hôpital Militaire de Nouakchott, Nouakchott, Mauritanie

**Keywords:** Traumatisme, cholédoque, péritonite biliaire, mortalité, Trauma, common bile duct, biliary peritonitis, mortality

## Abstract

La section traumatique du cholédoque est un phénomène extrêmement rare surtout lorsqu'elle est isolée. Habituellement, elle survient suite à une chirurgie des voies biliaires notamment laparoscopique. Cette lésion est souvent associée à une ou plusieurs lésions des organes de voisinage. Nous rapportons un cas de section isolée du cholédoque suite à un traumatisme abdominal chez un enfant âgé de 5 ans pris en charge dans notre service au Centre Hospitalier Mère et Enfant de Nouakchott. L'exploration radiologique n'était concluante. Une laparotomie exploratrice faite devant un tableau chirurgical a révélé une péritonite généralisée d'origine biliaire secondaire à une plaie du bas cholédoque. Le traitement d'urgence était une toilette avec une dérivation biliaire externe suivie à un mois après par une dérivation bilio-digestive. La complication la plus redoutable de section de cholédoque est la péritonite biliaire. La morbidité postopératoire précoce est de l'ordre de 20 à 30% dans la littérature alors que la mortalité est de 0 à 2%. La section traumatique isolée du cholédoque chez l'enfant est une lésion dont le tableau clinique et la conduite thérapeutique doivent être connus afin de diminuer la morbi-mortalité. La prise charge doit être multidisciplinaire impliquant le chirurgien pédiatre, le radiologue et le réanimateur anesthésiste.

## Abstract (en)

Traumatic injury of the common bile duct is extremely rare, especially when it is isolated. It usually occurs after laparoscopic biliary tract surgery. This lesion is often associated with one or multiple lesions of the neighborhood organs. We report a case of isolated injury of the common bile duct following abdominal trauma in a 5-year old child treated in our Department at the Mother-Child Hospital in Nouakchott. Radiological exploration didn't provide a conclusive diagnosis. Exploratory laparoscopy was performed because surgery had revealed diffuse biliary peritonitis secondary to a lesion at the bottom of the common bile duct. Emergency treatment was based on lavage with external biliary drainage followed, after a month, by bilio-digestive anastomosis. Life-threatening complication of common bile duct injuries is biliary peritonitis. In the literature, early postoperative morbidity is 20-30% while mortality rate is 0-2%. Isolated traumatic injury of the common bile duct in children is a lesion whose clinical course and therapy should be known in order to decrease morbi-mortality. Treatment is based on a multidisciplinary approach involving the pediatric surgeon, the radiologist and the resuscitating anaesthesiologist.

## Introduction

Les lésions des voies biliaires extrahépatiques suite à un traumatisme abdominal fermé sont une entité extrêmement rare. La vésicule biliaire est souvent l'organe le plus touché par ses traumatismes [1]. Habituellement ce traumatisme est associé à une lésion des organes de voisinage à savoir le foie, le pancréas, le duodénum et la rate [2, 3]. Ces lésions sont généralement méconnues et la laparotomie exploratrice reste le moyen le plus efficace pour identifier cette lésion dans les pays émergents. Nous rapportons un cas d'une lésion isolée du cholédoque suite à un traumatisme abdominal minime avec une revue de la littérature.

## Patient et observation

**Figure 1:**
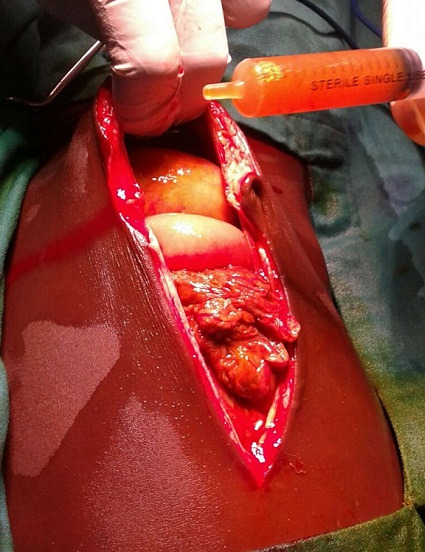
Péritonite biliaire avec épanchement bilieux abondant

Il s'agit d'une patiente âgée de 5 ans, victime d'un accident domestique: chute d'une charrette sur l'abdomen. Elle a consulté à notre urgence au 3^e^ jour de l'accident pour une douleur abdominale de l'hypochondre droit (HCD) isolée, l'état hémodynamique était bon. L'hémoglobine était à 10g/dl, le bilan pancréatique n'a pas été fait. L'échographie abdominale avait montré un épanchement de faible abondance dans le Douglass. Devant ce tableau l'enfant a été libéré sous traitement antalgique. Le dixième jour du traumatisme cette fille a été hospitalisée dans tableau de péritonite généralisée avec des signes de choc ayant nécessité un remplissage et une mise en place d'une triple antibiothérapie en urgence. En per-opératoire on avait trouvé une péritonite biliaire (Figure 1) par rupture du bas cholédoque, la vésicule biliaire, le foie, le duodénum, la rate et les deux reins sont intactes et les voies biliaires n'étaient pas dilatées. L'enfant a eu une toilette péritonéale abondante avec du sérum physiologique associé à un drainage biliaire externe par une sonde naso-gastrique N° 6 et un drainage péritonéal. Les suites opératoires immédiates ont été simples, marquées par la sortie de la réanimation au 4^e^ jour, une alimentation au 4^e^ jour après la reprise de transit et une ablation des fils au 10^e^ jour post opératoire. Dans le but de dilater les voies biliaires, cette fille avait subi une série de manœuvre de clampage de la sonde de drainage biliaire pendant 48h au cours desquels la fille avait fait un syndrome de cholestase clinique franc suivi de déclampage et ceci à partir du 15^e^ jour post opératoire. Après 15 jours, l'échographie abdominale a montré un canal hépatique propre dilaté à 7 mm. L'enfant a eu une dérivation bilio-digestive sur une anse en Y avec suites simples.

## Discussion

Le traumatisme du cholédoque est rare, en dehors des causes chirurgicales qui sont habituelles [2], le mécanisme le plus fréquent chez l'enfant est le coup épigastrique de guidon qui engendre un écrasement du cholédoque contre le rachis lombaire [3]. Dans ce cas la lésion du cholédoque est souvent associée à une lésion des organes intimes de voisinage à savoir le duodénum, le pancréas et le foie, et parfois le tronc cœliaque et les gros vaisseaux (l'aorte abdominale et la veine cave inferieure) [2, 3]. Dans notre cas le mécanisme était un coup sur l'épigastre par la barre de la charrette. L'examen clinique est souvent pauvre, une lésion ecchymotique ou pétéchiale parfois constatée dans la région épigastrique de l'enfant associée ou non à des douleurs abdominales modérées; dans les cas où la prise en charge était tardive, le patient vient dans un tableau de péritonite [2, 4] comme dans notre cas. Les fuites biliaires après traumatisme abdominal sont rares, elles surviennent généralement tardivement dans les trois semaines après le traumatisme [5]. L'échographie et le scanner [6, 7] sont utilisés en première intention dans la détection des fuites biliaires. Ils montrent le plus souvent un épanchement péritonéal de faible à moyenne abondance associé parfois à des lésions des organes de voisinage. D'après Barkun *et al*. la sensibilité du scanner (95%) est plus élevée que celle de l'échographie (75%) [8], mais ces examens manquent de spécificité. La cholangioIRM (CIRM) est une technique non invasive permettant de manière indirecte de détecter une fuite biliaire par la présence d'une collection. Sa sensibilité et sa spécificité pour la détection d'anomalies des voies biliaires sont estimées à 95% pour la sensibilité et 85% pour la spécificité [9]. Dans notre cas seule l'échographie abdominale été faite à cause du contexte d'urgence et elle n'avait pas apporté de diagnostic et elle n'a pas montré de lésions des organes avoisinants.

Le traitement de la péritonite biliaire due aux lésions traumatiques biliaires est médico-chirurgical et il doit être conduit en urgence. Ce traitement a été très bien discuté dans la littérature avec beaucoup de controverse [10, 11] mais le but commun est de maitriser l'inflammation péritonéale due au contact de la bile avec le péritoine et de permettre un écoulement facile de la bile dans le tube digestif. Le volet médical de ce traitement consiste à une réanimation avec un remplissage vasculaire associée à une triple antibiothérapie. En ce qui concerne le volet chirurgical qui doit commencer par un traitement endoscopique ou radiologique percutané en cas de disponibilité [12], à défaut de ce moyen, le traitement consiste premièrement à une toilette péritonéale abondante suivie d'un drainage externe des voies biliaires, habituellement par un drain de Kehr pendant quelques semaines. Le traitement définitif diffère selon la localisation et l'étendue de la lésion et selon les habitudes du chirurgien. L'anastomose termino-teminale est physiologique et elle est indiquée si la lésion siège sur la voie biliaire extra hépatique avec une perte de substance biliaire inférieure à 2cm chez l'adulte [4], mais cette technique nécessite une mobilisation du duodénum et de la tête du pancréas selon le manœuvre de Kocher pour diminuer la tension sur l'anastomose. Si l'écart est important et l'anastomose biliaire termino-terminale n'est pas possible à cause de la tension, le traitement consiste à une dérivation bilio-digestive cholédocho-jejunostomie selon Roux sur une anse en Y [2, 4, 13]. Cette dérivation est de plus en plus difficile et sera soldée à des complications type de sténose ou de fistule si la voie biliaire est fine ce qui était le cas de notre patiente. Pour augmenter le diamètre du cholédoque on a procédé à une dilatation iatrogène en clampant la sonde de drainage pendant 48h puis déclampage pendant 24h. A noter qu'à chaque fois qu'on clampait la sonde de drainage, la fille faisait un syndrome de cholestase qui disparait rapidement après le déclampage. Cette manœuvre a permis après 15 jours d'avoir un calibre du canal hépatique commun de 7 mm ce qui a facilité la dérivation bilio-digestive.

La gravité de cette affection est réellement débattue dans la littérature, selon la plupart des auteurs, le taux de morbidité postopératoire précoce est de 20 à 30% et le taux de mortalité de 0 à 2% [13-15]. Plusieurs complications ont été décrites après la chirurgie réparatrice des lésions des voies biliaires à savoir, les suppurations de la paroi, l'éventration, l'angiocholite, les abcès profonds, les sténoses anastomotiques, les sepsis et les défaillances multi viscérales [14, 16]. Quant à la péritonite biliaire la mortalité rapportée par des séries anciennes était très élevée entre 30 et 71% [16, 17], Tochi *et al*. rapporte un taux de mortalité à 7,7% [1]. La preuve d'un traitement chirurgical réussi est l'absence de sténose de l'anastomose biliaire. Dans les centres de référence, un résultat positif après réparation chirurgicale de la lésion biliaire est observé chez 70 à 90% des patients [16]. Les deux tiers (65%) des sténoses biliaires récurrentes se développent dans les 2 à 3 ans suivant la reconstruction, 80% dans les 5 ans et 90% dans les 7 ans [18]. Les sténoses récidivantes 10 ans après l'intervention chirurgicale sont également décrites dans la littérature [13]. Une durée de suivi nécessaire pour évaluer les résultats à long terme est de 2 à 5 ans [13, 15, 19]. Certains auteurs recommandent 10 à 20 ans d'observation [20].

## Conclusion

La section isolée du cholédoque chez l'enfant est une pathologie rare, elle doit être évoquée devant tout traumatisme abdominal. L'exploration radiologique n'a pas de grand apport au début du tableau clinique. La complication redoutable est la péritonite biliaire qui est mortelle, le traitement consiste à une dérivation bilio-digestive qui se déroule habituellement en deux temps. Le pronostic est mauvais si la prise en charge est tardive.

## Conflits d’intérêts

Les auteurs ne déclarent aucun conflit d'intérêts.
